# ROS-generating TiO_2_ nanoparticles for non-invasive sonodynamic therapy of cancer

**DOI:** 10.1038/srep23200

**Published:** 2016-03-21

**Authors:** Dong Gil You, V. G. Deepagan, Wooram Um, Sangmin Jeon, Sejin Son, Hyeyoun Chang, Hwa In Yoon, Yong Woo Cho, Maggie Swierczewska, Seulki Lee, Martin G. Pomper, Ick Chan Kwon, Kwangmeyung Kim, Jae Hyung Park

**Affiliations:** 1 School of Chemical Engineering, Sungkyunkwan University, Suwon 440-746, Republic of Korea; 2 Center for Theragnosis, Korea Institute of Science and Technology, 39-1 Hawolgok-dong, Seongbuk-gu, Seoul 136-791, Republic of Korea; 3 Department of Polymer Science and Engineering, Sungkyunkwan University, Suwon 440-746, Republic of Korea; 4 Samsung Advance Institute for Health Sciences and Technology, Sungkyunkwan University, Suwon 440-746, Republic of Korea; 5 Korea University of Science and Technology, 113 Gwahangno, Yuseong-gu, Daejeon 305-333, Republic of Korea; 6 Department of Chemical Engineering, Hanyang University, Ansan 426-791, Republic of Korea; 7 The Russell H. Morgan Department of Radiology and Radiological Science, Johns Hopkins School, Baltimore, Maryland 21287-0006, United States

## Abstract

The non-invasive photodynamic therapy has been limited to treat superficial tumours, primarily ascribed to poor tissue penetration of light as the energy source. Herein, we designed a long-circulating hydrophilized titanium dioxide nanoparticle (HTiO_2_ NP) that can be activated by ultrasound to generate reactive oxygen species (ROS). When administered systemically to mice, HTiO_2_ NPs effectively suppressed the growth of superficial tumours after ultrasound treatments. In tumour tissue, the levels of proinflammatory cytokines were elevated several fold and intense vascular damage was observed. Notably, ultrasound treatments with HTiO_2_ NPs also suppressed the growth of deeply located liver tumours at least 15-fold, compared to animals without ultrasound treatments. This study provides the first demonstration of the feasibility of using HTiO_2_ NPs as sensitizers for sonodynamic therapy *in vivo*.

Conventional cancer treatment modalities such as chemotherapy and surgery have several limitations including adverse drug reactions, impairment of the host immune system, and poor patient compliance^1^,^2^. In recent years, reactive oxygen species (ROS)-mediated cancer treatment, referred to as photodynamic therapy (PDT), has emerged as a potential alternative because of its minimal invasiveness and improved site-specific action^3^. Because of their high reactivity and very short half-life (<0.04 μs), ROS are effective only in close proximity (<0.02 μm) to the production site^4^. Furthermore, ROS upregulate the level of proinflammatory cytokines and leukocyte chemoattractants, which enhance the host immune response against tumours^5^,^6^,^7^. Clinical applications of PDT, however, have been hindered by the fact that photosensitizers produce ROS in the presence of light, which cannot reach deep tissues in the body^8^. Although considerable efforts have been made to improve the penetration depth of light using a near-infrared (NIR) pulsed laser, radiative losses and skin absorption of NIR light still pose a major challenge^9^. To overcome these issues, it is necessary to develop a new system that can generate ROS in deep tissue for effective cancer therapy.

Ultrasound (US) can penetrate much deeper in biological tissue because it is non-radiative and has a low tissue attenuation coefficient^10^. Although it is widely used for diagnostic imaging, US has recently emerged as a therapeutic modality in combination with drugs for various intractable diseases including cancer, diabetes, stroke, and cardiomyopathy^11^. In particular, it is a highly useful tool for controlling the physicochemical properties of nanoparticles (NPs) at pathologic sites for therapeutic applications^12^,^13^. The ability of US to activate some sensitizers such as porphyrin, and 5-aminolevulinic acid has led researchers to consider sonodynamic therapy (SDT) as a possible alternative to light-based PDT^14^,^15^.

Titanium dioxide nanoparticles (TiO_2_ NPs) are nontoxic to mammalian cells because they are chemically inert and stable under physiological conditions^16^,^17^. Because of their biocompatibility, TiO_2_ NPs have been investigated as potential carriers for anticancer drugs and genes^18^,^19^. In addition, photocatalytic traits of TiO_2_ NPs make them attractive as photosensitizers for PDT. Their applications for PDT, however, are limited because their high bandgap (~3.2 eV) allows ROS generation in the presence of ultraviolet light, which has poor penetration into biological tissue and can cause DNA damage. Efforts have been made to use the upconversion NIR system to activate TiO_2_ NPs *in vivo* to generate ROS^20^. Unfortunately, this method has similar problems to conventional PDT. Owing to the non-radiative nature of US, SDT seems to be a more viable option for activating TiO_2_ NPs to generate ROS^21^. Nonetheless, *in vivo* cancer therapy using TiO_2_ NPs and SDT has not been investigated, primarily ascribed to their poor tumour targetability and instability in biological fluids.

Here, we report on hydrophilized TiO_2_ NPs (HTiO_2_ NPs) that produce ROS *in vivo* when activated by US to eradicate tumours. The working mechanism of HTiO_2_ NPs *in vivo* is illustrated in Fig. 1a. As a hydrophilic polymer, we chose carboxymethyl dextran (CMD) which was chemically anchored on the surface of TiO_2_ NPs to prepare HTiO_2_ NPs. CMD is widely used as a hydrophilic compartment in NPs and drug conjugates to prolong systemic circulation^22^. Unlike other hydrophilic polymers such as polyoxazoline and polyethylene glycol, CMD has multiple sites for chemical modification. In addition to being non-immunogenic, polyanionic CMD shows an extended blood circulation time compared to its non-ionic counterpart^23^. Previously, we also demonstrated that CMD-based NPs have excellent tumour-homing abilities because of their prolonged circulation *in vivo*^24^,^25^. In this study, we demonstrated that HTiO_2_ NP-based SDT generate a high level of ROS both *in vitro* and *in vivo*. In addition, we verified the upregulation of proinflammatory cytokines and interleukins (ILs) in tumour tissue after SDT. We also showed that SDT can cause destruction of the tumour microvasculature, which in turn leads to infarction. Moreover, we have successfully demonstrated for the first time that SDT can supress growth of a deeply located tumour.

## Results

### Physicochemical characterization

**Figure 1:**
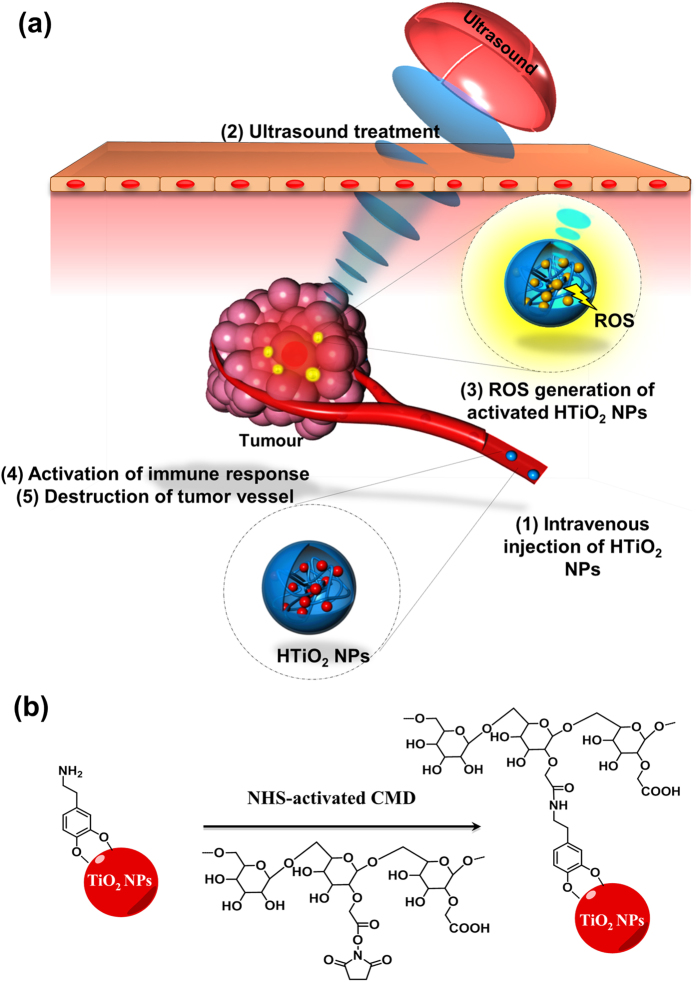
Schematic illustration of SDT using HTiO_2_ NPs. (**a**) After systemic administration of HTiO_2_ NPs, they reach the tumor site by the enhanced permeation and retention effect. The NPs can be activated to produce ROS when exposed to ultrasound, which may enhance immune responses and destroy tumor microvasculature. (**b**) Surface modification of TiO_2_ NPs.

**Figure 2:**
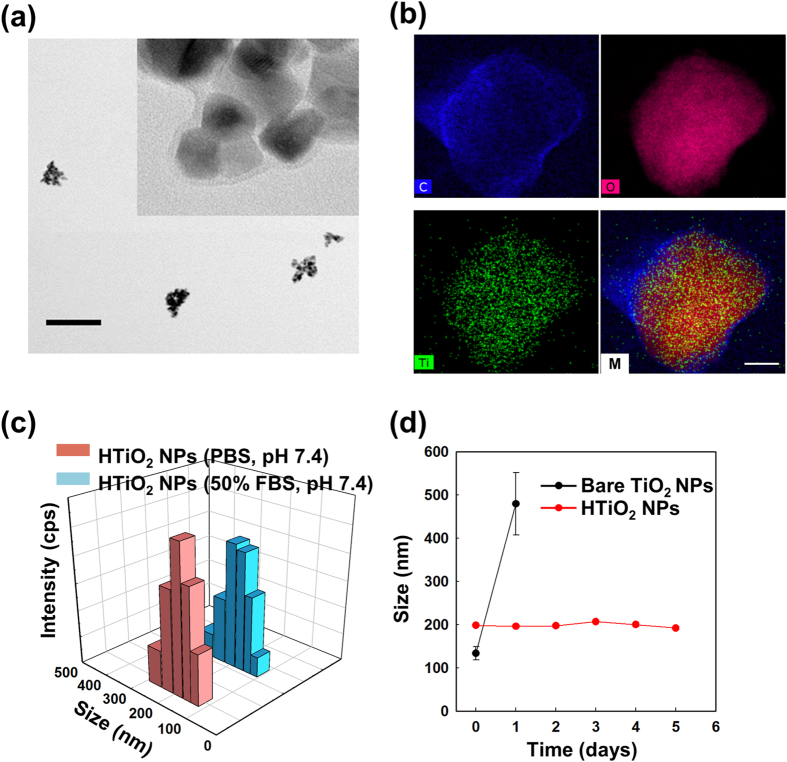
Characterization of HTiO_2_ NPs. (**a**) TEM images of HTiO_2_ NPs. The scale bar is for 500 nm. (**b**) EDS mapping images of HTiO_2_ NPs to show robust coating of CMD on the TiO_2_ NPs. The scale bar is for 90 nm. (**c**) Size distribution of HTiO_2_ NPs in PBS and in serum conditions. **(d)** Changes in sizes of bare and HTiO_2_ NPs as a function of time (PBS, pH 7.4).

HTiO_2_ NPs were prepared by chemical modification of dopamine-decorated TiO_2_ NPs with CMD (Fig. 1b). FT-IR and energy-dispersive X-ray spectroscopy (EDS) data (Fig. S1a,b) confirmed the formation of an amide bond and the presence of CMD on the surface of TiO_2_ NPs. TEM and EDS mapping images implied that the TiO_2_ NPs were totally covered by the CMD polymer layer (Fig. 2a,b). The prepared HTiO_2_ NPs had a hydrodynamic radius of 198.33 ± 2.6 nm with a zeta potential value of −15.8 mV (Fig. 2c). Also, no significant changes in the size of the HTiO_2_ NPs were observed, even after 24-h incubation in the presence of 50% FBS. The HTiO_2_ NPs showed good stability in PBS (pH 7.4) for at least 5 days, whereas bare TiO_2_ NPs aggregated within one day (Fig. 2d). Thereafter, to evaluate the deforming ability and flexibility of HTiO_2_ NPs, the filtration test was carried out by passing Cy5.5-labeled HTiO_2_ NPs through a series of syringe filters (0.8 μm, 0.45 μm, and 0.2 μm). In this experiment, the Cy5.5-labeled polystyrene NPs (200 nm in diameter) served as a control (Fig. S2a,b). It was evident that HTiO_2_ NPs could pass through all filters because of their deformability, whereas a substantial amount of polystyrene NPs was unable to pass through the syringe filters with the pore size less than 0.45 μm. The cytotoxicity of the HTiO_2_ NPs was evaluated by the methylthiazol tetrazolium (MTT) assay and flow cytometric analysis (Fig. S3a,b). No significant cytotoxicity was observed when SCC7 and NIH3T3 cells were exposed to the HTiO_2_ NPs.

### Tumor-homing ability of HTiO_2_ NPs

**Figure 3:**
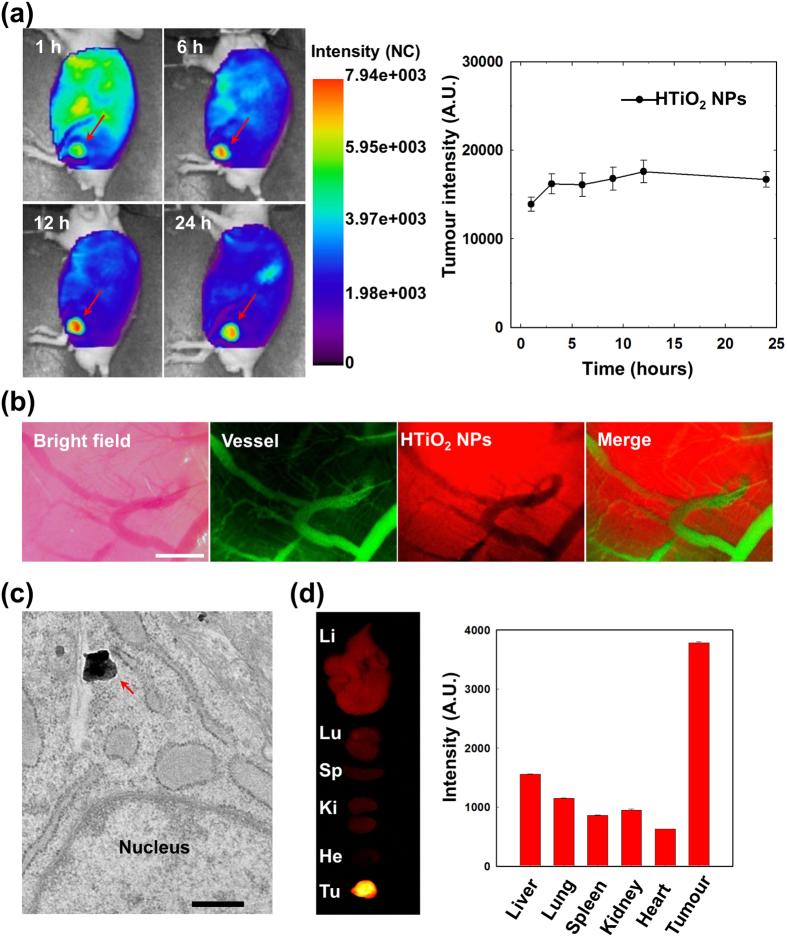
*In vivo* biodistribution of HTiO_2_ NPs in SCC7 tumour-bearing mice. (**a**) Time-dependent whole-body distribution of Cy5.5-labeled HTiO_2_ NPs in SCC7 tumor-bearing mice after intravenous administration. The fluorescence intensity reached a maximum within 12 h. Error bars represent the standard deviation (*n* = 3). (**b**) *In vivo* fluorescent images of tumor tissues. Vessels (green) and HTiO_2_ NPs (red) were observed 12 h after intravenous injection. Scale bar, 1,000 μm. (**c**) *Ex vivo* TEM images of tumor tissues 12 h after intravenous injection. The arrow indicates the HTiO_2_ NPs (scale bar, 300 nm). (**d**) *Ex vivo* fluorescence images of organs and tumor 24 h post injection. Quantification of *ex vivo* fluorescence intensity of organs and tumor. Error bars in the graphs represent standard deviation (*n* = 3).

The *in vivo* biodistribution and tumour-homing ability of HTiO_2_ NPs were monitored after systemic administration of Cy5.5-labeled HTiO_2_ NPs into SCC7 tumour-bearing mice using a real-time NIR fluorescence imaging technique (Fig. 3a). During the initial hour, the fluorescence signal was detected throughout the body, presumably reflecting circulation of HTiO_2_ NPs in the bloodstream. Interestingly, the strongest fluorescent signal was observed at the tumour site, which gradually increased until 12 h, suggesting the high tumour targetability of HTiO_2_ NPs. At 24 h, the tumour region could be clearly demarcated from the surrounding tissue. Magnification of tumour tissue in the live animal model revealed that the HTiO_2_ NPs (red) had effectively escaped from the blood vessel (green), followed by deep tissue penetration (Fig. 3b). Furthermore, when a section of excised tumour tissue was examined under TEM, HTiO_2_ NPs were readily detected at the intracellular level (Fig. 3c). The *ex vivo* tissue images of major organs, obtained from tumour-bearing mice 24 h post injection, indicated selective accumulation of HTiO_2_ NPs in the tumour tissue. From quantification analysis (Fig. 3d), the signal intensity of HTiO_2_ NP at the tumour tissue was 2.4− to 6.0-fold higher than in major organs.

### *In vitro* ROS generation by HTiO_2_ NPs and cytotoxicity

**Figure 4:**
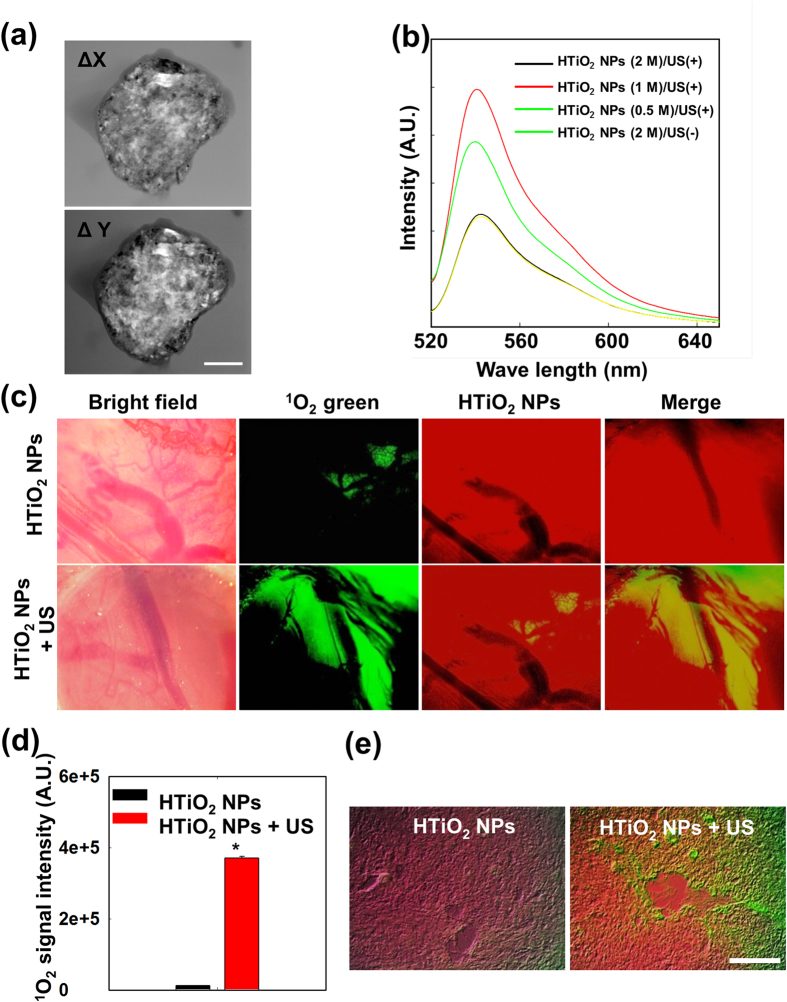
ROS genration ability of HTiO_2_ NPs. (**a**) Phase contrast imaging of electron field distribution and ronchigram for HTiO_2_ NPs. The scale bar is for 90 nm. (**b**) HTiO_2_ NPs dose-dependent ^1^O_2_ generation after ultrasound treatment *in v*i*tro*. The sample without ultrasound treatment serves as a baseline. (**c**) *In vivo* fluorescent imaging of the tumour site to observe ^1^O_2_ (green) and HTiO_2_ NP (red) 12 h after SDT. Scale bar, 1,000 μm. (**d**) Quantification of ^1^O_2_ fluorescence signal intensity. The signal intensities of the red area increased after SDT (*p < 0.001). Error bars in the graphs represent standard error (*n* = 3). (**e**) *Ex vivo* fluorescence images of tumor tissue to observe ^1^O_2_ (green) and HTiO_2_ NPs (red) 12 h after SDT. Scale bar, 200 μm.

The distribution of the electron field around the HTiO_2_ NPs is important as it directly correlates with the efficacy of ROS generation. The bright and dark patches on the ronchigram, obtained from differential phase contrast (DPC) images, confirms the presence of electron fields in and around HTiO_2_ NPs (Fig. 4a). To observe *in vitro* ROS generation by HTiO_2_ NPs, their suspension in PBS (pH 7.4) containing diphenylisobenzofuran (DPBF) or ^1^O_2_-sensor green was placed in an agarose mould and then treated with US. The result showed that the amount of ROS generated by HTiO_2_ NPs increased with the US exposure time (Fig. S4a). The rate constant value for ^1^O_2_ generation was determined to be 0.00105 s^−1^ (Fig. S4b). In addition, HTiO_2_ NPs generated ^1^O_2_ in a dose-dependent manner (Fig. 4b).

### *In vivo* ROS generation by HTiO_2_ NPs upon US treatment

To evaluate ^1^O_2_ generation *in vivo*, fluorescence images of the tumour tissue were obtained 12 h after systemic administration of Cy5.5-labeled HTiO_2_ NPs. The red fluorescence in the tumour image shows that the HTiO_2_ NPs were distributed evenly in US-treated and non-treated animal groups. Without US treatment, the ^1^O_2_ signal (green) was rarely detected in the tumour tissue. Interestingly, in the US-treated mouse, strong fluorescence was observed mostly around blood vessels in tumour tissue (Fig. 4c). Quantitative analysis indicated that US-treated tumour tissue contained 29.7-fold more ^1^O_2_ molecules than non-treated tissue (Fig. 4d). This observation clearly illustrates that the HTiO_2_ NPs could generate ^1^O_2_ only when treated with US. In addition, visualization of sections of excised tumour under a fluorescent microscope (Fig. 4e) was consistent with the *in vivo* fluorescence image: Red fluorescence (from the HTiO_2_ NPs) could be seen throughout the tumour section, whereas green fluorescence (from ^1^O_2_-sensor green) could be seen only in US-treated tumours.

### Antitumor efficacy of HTiO_2_ NPs for superficial tumor

**Figure 5:**
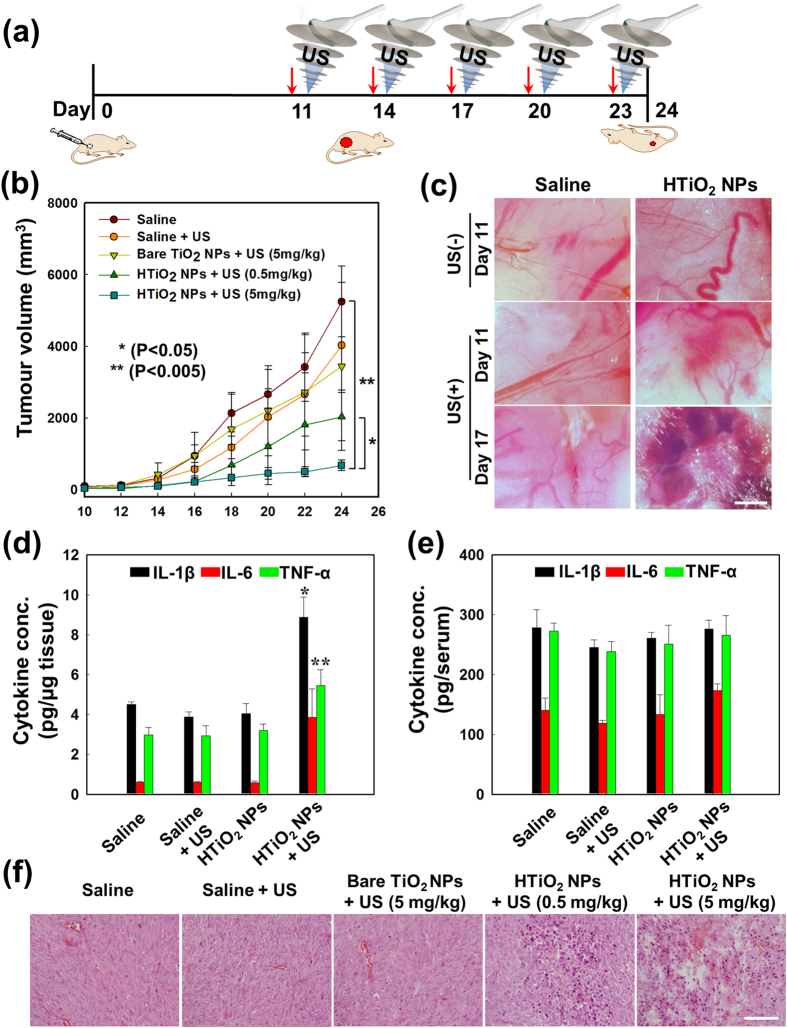
Antitumour efficacy of HTiO_2_ NPs in SCC7 tumour-bearing mice. (**a**) The treatment regimen of SDT. The red arrow represents systemic administration of HTiO_2_ NPs. (**b**) Changes in tumor volume for each treatment group (*p < 0.05, **p < 0.005 calculated by one-way ANOVA test). Error bars in the graphs represent standard deviation (*n* = 5). (**c**) Bright-field images of tumor vasculature after SDT with US. Scale bar, 1,000 μm. (**d**) SDT-induced immune response to tumor. Cytokine levels of tumor tissues after SDT. The error bars represent standard deviation (*n* = 5) with *p < 0.001, **p < 0.005 (calculated by one-way ANOVA test). (**e**) Cytokine levels in serum of mice after SDT. (**f**) H&E staining images of tumor tissue from each treatment group. Scale bar, 200 μm.

The antitumour efficacy in SCC7 tumour-bearing C3H/HeN mice after intravenous administration of HTiO_2_ NPs was monitored for 24 days (Fig. 5a). For this experiment, the flank tumour model was prepared by subcutaneous injection of the tumour cells. As anticipated, the control group treated only with saline exhibited a rapid increase in tumour size up to a volume of 2,000 mm^3^ within 18 days (Fig. 5b). Similarly, animals treated with saline and US showed an increase in tumour volume up to 2,000 mm^3^ within 20 days. The US treatment also failed to suppress tumour growth in animals that received bare TiO_2_ NPs. This is primarily ascribed to the tumour-homing ability of bare TiO_2_ NPs with poor stability, as shown in Fig. 2d. On the other hand, a significant reduction in tumour size (p < 0.005) was observed in mice treated with HTiO_2_ NPs and US compared with controls. Likewise, a dose-dependent reduction in tumour volume was observed between the US-treated animals with HTiO_2_ NPs (0.5 mg/kg) and HTiO_2_ NPs (5 mg/kg) (p < 0.05). Of note, HTiO_2_ NPs (5 mg/kg) plus US treatment suppressed tumour volume to below 700 mm^3^ over 24 days. It implies that there is the correlation between tumour regression and ROS generation by US-activated HTiO_2_ NPs. In addition, a substantial amount of vascular damage was observed in tumours treated with HTiO_2_ NPs (5 mg/kg) and US from day 17 (Fig. 5c). The extent of damage was increased with the treatment progress. Here again, minimal damage was recorded in the vasculature after treatment with either US or HTiO_2_ NPs alone.

Motivated by these findings, we further evaluated the levels of proinflammatory cytokines in the tumour and the systemic circulation. Levels of IL-6, IL-1β, and tumour necrosis factor-α (TNF-α) in tumour tissue were 6.41−, 1.97−, and 1.83-fold higher in mice treated with HTiO_2_ NPs and US than in saline-treated control animals (Fig. 5d). However, there was no significant difference in the level of cytokines among the tumours treated with saline, US, or HTiO_2_ NPs. These results suggest that the elevated level of cytokines is due to ROS generated by the SDT, since ROS are known to increase levels of the proinflammatory cytokines^3^. Interestingly, the levels of IL-6, IL-1β, and TNF-α in systemic circulation did not show significant differences among the experimental groups (Fig. 5e). This clearly demonstrates the site-specific induction of cytokines in the target site by the SDT. These results suggest that SDT performed with HTiO_2_ NPs has the beneficial effects of PDT, such as enhancing the production of proinflammatory cytokines and inducing destruction of tumour blood vessels. Histologic staining confirmed that the tumours treated with HTiO_2_ NPs (5 mg/kg) showed enhanced cell death compared with other treatment groups (Fig. 5f). Finally, systemic toxicity, assessed by histologic analysis of major organs after treatment (Fig. S5), revealed that mice treated with HTiO_2_ NPs did not show any histopathologic changes in major organs.

### Antitumor efficacy of deeply located tumor in the liver

**Figure 6:**
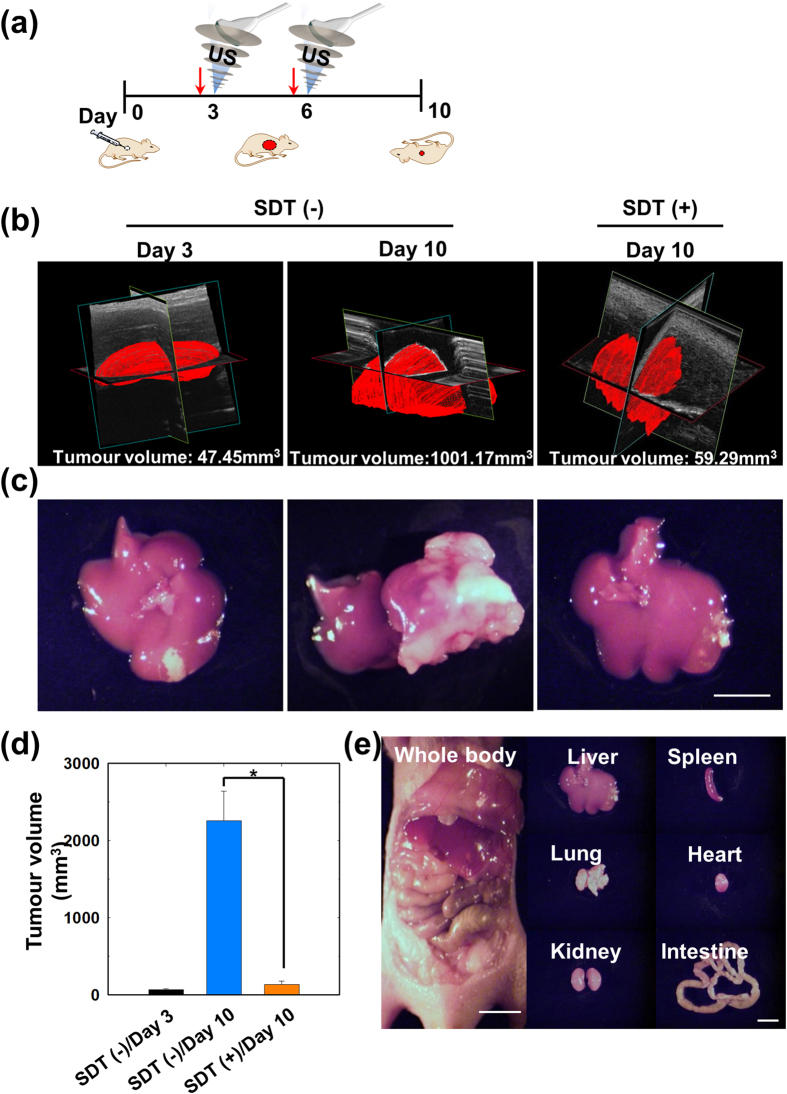
Antitumour efficacy of HTiO_2_ NPs in the liver tumour model. (**a**) The treatment regimen of SDT. The red arrow represents systemic administration of HTiO_2_ NPs. **(b)** *In vivo* ultrasound 3D-rendered images of liver tumour after SDT. (**c**) *Ex vivo* bright-field images of liver tumour after SDT. (**d**) Tumour volume for each treatment group. Error bars in the graphs represent standard deviation (*n* = 5) with *p < 0.001. (**e**) Bright-field images of major organs after SDT in a liver tumour model (Scale bar, 1 cm).

Lastly, we studied the effectiveness of HTiO_2_ NPs-based SDT for treating tumours in deeper regions. A liver tumour model was established by injecting SCC7 cells into the left lobe of the liver in nude mice. The *in vivo* biodistribution image recorded at 3 h post systemic administration of Cy5.5-labeled HTiO_2_ NPs showed the strong fluorescence intensity in the liver, especially from the left lobe (Fig. S6a). The *ex vivo* image of the liver confirmed that the signal intensity was indeed from the tumour region (Fig. S6b). Therefore, it was evident that the HTiO_2_ NPs preferentially accumulated in the liver tumour rather than in healthy liver tissue. The therapeutic effect was analysed by comparing animal groups with and without US treatment at 24 h after intraveneous administration of HTiO_2_ NPs (Fig. 6a). From the ultrasound 3D visualization of the tumour, it was evident that US-treated animals showed a substantial suppression of tumour growth (Fig. 6b). In non-treated mice, the tumour volume rapidly increased from 47.45 mm^3^ on day 3 to 1,001.17 mm^3^ on day 10. However, in SDT-treated mice, the tumour volume was only 59.29 mm^3^ on day 10. In fact, the actual tumour volume of non-treated mice were larger than the size detected by 3D ultrasound visualization because the tumour volume exceeded the maximum field of observation. *Ex vivo* analysis revealed a 16.9-fold difference in tumour volume (Fig. 6c,d) between control and US-treated groups. Furthermore, macroscopic analysis of the major organs of animals treated with US showed no sign of metastasis (Fig. 6e). Overall, *in vitro* and *in vivo* analyses implicate HTiO_2_ NPs as a potential sonosensitizer for effective cancer treatment by SDT.

## Discussion

Although efforts have been made to develop NPs for targeted delivery of photosensitizers^26^, PDT is limited to superficial tumours because of poor tissue penetration of light as the energy source. In order to activate the photosensitizer in deep sites, optical fibres have been used to deliver light to the targets^27^. These optical fibres were often custom made to treat specific types of cancer, whereas it would be difficult to treat large tumours in this manner. In contrast to light, US is delivered much deeper into the body^10^,^28^. Also, US can be used to generate ROS *in situ* in presence of sonosensitizers^29^. In the current study, we investigated the feasibility of long-circulating HTiO_2_ NP-based SDT as a potential alternative to PDT. Compared to conventional porphyrin-based sonosensitizers which are quickly degraded under oxidizing conditions, HTiO_2_ NPs may have high stability because inorganic TiO_2_ is resistant to degradation by ROS. The hydrophilic CMD coat on the HTiO_2_ NPs enhanced the stability and increased the blood circulation time. The hydrophilic nature of CMD imparts stealth characteristics to HTiO_2_ NPs and provides a flexible surface that is critical for extravasation from the leaky vasculature of the tumour. The DPC image of the NPs confirmed the presence of an electron field even after surface modification with CMD. These electrons on the HTiO_2_ surface might play an important role in generating ROS when exposed to US.

Although the mechanism of ROS generation during US treatment is not fully understood, sonoluminescence is believed to be a key phenomenon to generate ROS^15^. We have demonstrated that HTiO_2_ NPs generate ROS *in vitro* when exposed to US in a dose-dependent manner (Fig. 3). In addition, *in vivo*^1^O_2_ mapping of the tumour showed a substantial increase in ROS level for US-treated tumours in the presence of HTiO_2_ NPs (Fig. 5). Despite the fact that the HTiO_2_ NPs penetrated deep into tumour tissue, the *in vivo* fluorescence image of ROS showed a stronger fluorescence around blood vessels than in deeper regions. This might reflect the higher *p*O_2_ level around blood vessels, which in turn fuels ^1^O_2_ production. Administration of HTiO_2_ NPs to the flank tumour model demonstrated that SDT suppressed the growth of tumours (Fig. 5b). It was revealed that regression of the tumour is due to the elevated level of ROS which eradicates the tumour cells as well as causes destruction of tumour blood vessels. ROS can induce blood stasis via platelet aggregation or directly damage the blood vessel by destroying the endothelial layer^30^,^31^. Furthermore, as in PDT, we have shown that SDT elevated the level of immuno-enhancers such as IL-1β, IL-6, and TNF-α. These chemoattractants are known to stimulate the maturation and function of granulocytes and macrophages^32^. SDT of liver tumour model with HTiO_2_ NPs proved that it could effectively suppress the growth of tumour in the deep site.

SDT with HTiO_2_ NPs could be used as an adjuvant therapy for high intensity-focused ultrasound (HIFU) treatment. In the clinic, HIFU is used to ablate tumours through hyperthermia as one of non-invasive surgeries. Complete eradication of tumour cells using HIFU relies significantly on precision and operational skills as microscopic recurrence of residual tumour tissue could occur, especially in poorly differentiated tumours and sites near anastomosis^33^. In order to increase therapeutic efficacy, several studies have demonstrated that the combination of HIFU and chemotherapy significantly reduce the risk of relapse^34^,^35^,^36^. However, this approach is seriously limited by the fact that anticancer drugs can suppress tumoural immune response. As we have proven that HTiO_2_ NPs prepared in this study upregulate proinflammatory cytokines, the immunomodulatory mechanism is not only preserved, but is even enhanced, by SDT. In summary, SDT using HTiO_2_ NPs with US is a promising therapeutic strategy that could open a new window in the fight against cancer.

## Methods

### Materials and reagents

CMD sodium salt (Mw = 10,000–20,000 Da), 1-ethyl-3-(3-dimethylaminopropyl)-carbodiimide·hydrochloride (EDC·HCl), *N*-hydroxysulfosuccinimide (NHS), dopamine·HCl, and anatase TiO_2_ NPs (25 nm in diameter) were purchased from Sigma Chemical Co. (St. Louis, MO, USA). The NIR dye, FCR-675 amine, was purchased from BioActs (Incheon, Korea). Cy5.5 was purchased from Amersham Biosciences (NJ, USA). Singlet oxygen sensor green reagent was purchased from Life Technologies Korea LLC (Seoul, Korea). The ELISA complete kit for mouse IL-6, IL-1β, and TNF-α was purchased from Komabiotech (Seoul, Korea). All other reagents were analytical grade. SCC7 and NIH3T3 cell lines were purchased from the American Type Culture Collection (ATCC, Rockville, MD, USA). For cell culture, RPMI-1640, DMEM, trypsin-EDTA, and fetal bovine serum (FBS) were purchased from Welgene Inc. (Daegu, Korea). All experimental involving live animals were carried out in accordance with the relevant laws and institutional guidelines of Sungkyunkwan University. The Sungkyunkwan University institutional committees have approved all the experimental protocols.

### Synthesis of HTiO_2_ NPs

TiO_2_ NPs were prepared in a two-step procedure in which dopamine was functionalized on the TiO_2_ NPs, followed by chemical conjugation of NHS-activated CMD.

Dopamine stock solution was prepared by dissolving 1.92 μmoles of dopamine·HCl in 10 ml of deionized water. Dopamine-decorated TiO_2_ NPs (D-TiO_2_ NPs) were prepared by dispersing 10 mg of TiO_2_ NPs (anatase) in 5 ml formamide and stirring vigorously. To this suspension, 100 μL of dopamine stock solution was added slowly and the resulting suspension was stirred for an additional 6 h. D-TiO_2_ NPs were purified by three rounds of centrifugation at 13,000 rpm and washing with formamide. Finally, the D-TiO_2_ NPs were suspended in 10 ml formamide.

For the activation of CMD, 200 mg of CMD was dissolved in 20 ml of formamide. After addition of 76.7 mg EDC and 57.5 mg of NHS, the solution was stirred overnight. 2 ml of D-TiO_2_ NPs solution was then added drop wise to the activated CMD solution and the reaction was allowed to continue for 10 h. The reaction was terminated by addition of 75 μl of 0.1 M NaOH. Byproducts and excess polymer were removed by dialysis against sodium borate buffer (pH 8.6) at 4 °C using a 50-kDa cut off membrane for 48 h with buffer changes every 6 h. The dialysate was sonicated for 10 seconds (Sonic Vibracell VCX 750, CT, USA) with 30% amplitude and filtered to remove the large aggregates. Finally, the suspension was filtered through a 0.8-μm filter and freeze-dried for future use. Cy5.5-conjugated NPs were formed by reaction of NHS-activated HTiO_2_ NPs with amine-functionalized Cy5.5. The Cy5.5-labeled NPs were purified by dialysis and freeze-dried for future use. All reactions were carried out in the dark to avoid photoactivation of TiO_2_ NPs.

### Cell culture

SCC7 (mouse squamous carcinoma cell line) and NIH3T3 (mouse embryonic fibroblast cell line) cells were cultured with RPMI 1640 and DMEM media respectively. All cell growth media were supplemented with 10% FBS, 100 U/mL penicillin, and 100 μg/mL streptomycin, and the cells were cultured at 37 °C in a humidified CO_2_ incubator.

### *In vitro* cytotoxicity assay

SCC7 and NIH3T3 cells were seeded in 96-well flat-bottomed plates at a density of 1 × 10^4^ cells/well and incubated for 24 h. The cells were washed twice with PBS, replenished with media containing various concentrations of the test samples, and incubated for a further 24 h. The cells were washed once with PBS and then 100 μl of basal media containing 10% MTT solution (5 mg/ml) was added and incubated for 3 h. After removal of the medium, the MTT crystals were dissolved in DMSO and the absorbance of each well was measured at 570 nm using a microplate reader (VERSA max, Molecular Devices Corp., Sunnyvale, CA, USA).

For flow cytometry analysis, SCC7 and NIH3T3 cells were seeded in 6-well plates at a density of 5 × 10^5^ cells per well. The cells were incubated to form a monolayer, washed with PBS twice, and replenished with media containing 100 μg/ml of the test sample. After incubation, the cells were stained with a LIVE/DEAD cell vitality assay kit (Life Technologies, CA, USA) and analysed by flow cytometry according to the manufacturer’s protocol.

### *In vivo* biodistribution of HTiO_2_ NPs

Tumour-bearing mice were prepared by subcutaneously injecting 80 μl of a suspension of 1 × 10^6^ SCC7 cells. After 14 days, HTiO_2_ NPs were injected into the tail vein of the mice at a dose of 5 mg/kg. The biodistribution was measured using the Explore Optix system (ART Advanced Research Technologies Inc., Montreal, Canada) with the laser set to an output power of 10 μW for 0.3 second per point. The tumour-targeting characteristics of HTiO_2_ NPs were evaluated by measuring the NIR fluorescence intensity at the tumour site (30 mm^2^). All data were calculated using the region of interest (ROI) function of Analysis Workstation software (ART Advanced Research Technologies Inc., Montreal, Canada).

To analyse the distribution of HTiO_2_ NPs in tumour tissue, the skin over the tumour of the mice was scraped off 12 h after intravenous injection of Cy5.5-labeled HTiO_2_ NPs (5 mg/kg). In order to visualize the tumour blood vessel, FITC-labeled dextran (10 mg/kg) was administered intravenously to the mice immediately before the measurement. NIR fluorescence images of the tumour were obtained using a small-animal imaging system (OV-100, Olympus, Center Valley, PA) with the green flurescenct protein (GFP) channel (λ_ex_ = 450–480 nm with λ_em_ 500–530 nm) and Cy5.5 channel (λ_ex_ = 620–650 nm with λ_em_ = 680–710 nm). After imaging, the mice were sacrificed and sections of tumour were processed for e*x vivo* transmission electron microscopy (TEM) imaging using a Cryo-TEM (FEI Tecnai F20, Oregon, USA).

Mice were sacrificed 24 h after intravenous injection of HTiO_2_ NPs (5 mg/kg) and NIR fluorescence images of dissected organs and tumours were obtained with a 12-bit CCD camera (Kodak Image Station 4000MM, New Haven, CT) equipped with a special C mount lens and Cy5.5 band pass emission filter (680–720 nm; Omega Optical). The tissue distribution of HTiO_2_ NPs was quantified by measuring NIR fluorescence intensity at the ROI.

### Histology

Major organs, including liver, heart, kidneys, and spleen, were excised from mice 24 h post injection. Organs were fixed in 3.7% neutral buffered formalin, processed into paraffin, sectioned into approximately 4-μm slices, and stained with hematoxylin and eosin (H&E). The samples were chosen at random and examined by bright-field microscopy (Olympus BX61 inverted microscope).

### *In vitro* ROS generation

The *in vitro* singlet oxygen generation by HTiO_2_ NPs was estimated by chemical oxidation of DPBF. Briefly, HTiO_2_ NPs (2 M) were exposed to ultrasound in the presence of DPBF (2 × 10^−5^ M). The chemical oxidation of DPBF was determined using the UV-vis spectrometer (G1103A, Agilent, USA) by measuring absorbance at 413 nm as a function of time. The rate constant value for ^1^O_2_ generation was calculated by the following equation [ln([DPBF]_t_/[DPFP]_0_) = −kt]^37^.

To detect ^1^O_2_ generation induced by HTiO_2_ NPs *in vitro*, 0.5 ml of HTiO_2_ NPs solution was added to 0.5 ml of 40 μM 2′–7′-dichlorofluorescin solution^38^. The resulting solution was transferred to a 3% (W/V) agarose gel mould and exposed to high intensity-focused US (VIFU-2000, Alpinion medical system, Seoul, Korea) for 330 seconds (power: 30 W, duty cycle: 10%, pulse repetition frequency: 1 Hz, X, Y interval: 2 mm). The fluorescence intensity of singlet oxygen was measured using a fluorescence spectrophotometer (F-7000, Hitachi, Tokyo, Japan).

### *In vivo*^1^O_2_ detection in tumour site

Mice were injected with 100 μl of ^1^O_2_ sensor green reagent (50 μM) intratumourally and with HTiO_2_ NPs (Ti 5 mg/kg) intravenously. The mice were treated with US 12 h after injection of HTiO2 NPs. The florescence image of ^1^O_2_ was measured 30 min after US treatment (power: 30 W, frequency: 1.5 MHz, duty cycle: 10%, pulse repetition frequency: 1 Hz, time: 30 seconds, interval: 2 mm). The target positions were exposed to US for 330 seconds. Fluorescence images of ^1^O_2_ and HTiO_2_ NPs in tumour tissues were obtained using a small-animal imaging system (OV-100, Olympus) with bright field, GFP channel (λ_ex_ = 450–480 nm with λ_em_ 500–530 nm), and Cy5.5 channel (λ_ex_ = 620–650 nm with λ_em_ = 680–710 nm). Quantification of fluorescence images was analysed by Image-pro plus (Media Cybernetics, USA). A portion of tumour tissue was then cryosectioned at 10-μm thickness and tumour sections were observed by fluorescence microscopy (IX81-ZDC, Olympus, Tokyo, Japan) using a GFP and Cy5.5 filter.

### Antitumour efficacy of SDT with HTiO_2_ NPs

The antitumour efficacy of HTiO_2_ NPs was determined by measuring tumour volume as a function of time. Tumour-bearing mice were prepared by injection of 80 μl of a suspension of 1 × 10^6^ SCC7 cells. When tumours reached a volume of 50–100 mm^3^, mice were divided into six groups: (a) saline, (b) saline + US, (c) bare TiO_2_ NPs (Ti 5 mg/kg) + US, (d) HTiO_2_ NPs (Ti 0.5 mg/kg) + US, and (e) HTiO_2_ NPs (Ti 5 mg/kg) + US. US treatment was conducted 12 h after systemic administration of HTiO_2_ NPs. Each treatment was given once every 3 days. The mice were treated by US in the pre-set condition (power: 30 W, frequency: 1.5 MHz, duty cycle: 10%, pulse repetition frequency: 1 Hz, time: 30 seconds, interval: 2 mm). The tumour tissues were sequentially exposed to US at 11 focal points for 300 seconds. Tumour volumes were calculated as **a** × **b**^2^ × 0.54, where **a** was the largest and **b** the smallest diameter. Histologic changes and apoptotic cells in tumour tissues were evaluated using H&E staining. Slides containing 5-μm-thick sections were prepared and observed using an Olympus BX51 microscope (Tokyo, Japan).

### Detection of tumour vessel destruction by SDT

*In vivo* vascular damage after SDT was analysed by following the same treatment regimen as that of antitumour efficacy of SDT with HTiO_2_ NPs (Fig. 5A). Mice were divided into two groups: (i) group 1 which was treated only with US and (b) group 2 which was treated with HTiO_2_ NPs (Ti 5 mg/kg) and US. The skin of mouse tumours was scraped off after SDT on day 11 and 17. The mice were exposed to US with an output power of 10 W. The tumour vessels were detected by bright-field imaging using a small-animal imaging system (OV-100, Olympus).

### Evaluation of cytokines using ELISA

Tumour tissues and serum samples were obtained 24 days after SDT. Mice were divided into four groups: (a) saline, (b) saline + US, (c) HTiO_2_ NPs (Ti 5 mg/kg), and (d) HTiO_2_ NPs (Ti 5 mg/kg) + US. IL-6, IL-1β, and TNF-α levels in tumour tissues and serum were determined using ELISA kits, according to the manufacturer’s instructions.

### *In vivo* biodistribution of HTIO_2_ NPs in the liver tumour model

To evaluate the tumour accumulation properties of HTiO_2_ NPs, the SCC7 liver tumour model was prepared by injection of 20 μl of a suspension of 3 × 10^5^ SCC7 cells into the liver tissue. After 5 days, HTiO_2_ NPs were injected into the tail vein at a dose of 5 mg/kg. The biodistribution images were obtained using the Explore Optix system. Laser power and exposure time were fixed at 10 μW and 0.3 seconds per point, respectively. The tumour-targeting characteristics of HTiO_2_ were evaluated by measuring the NIR fluorescence intensity at the tumour site (30 mm^2^). All data were calculated using the ROI function of Analysis Workstation software. Mice were sacrificed 6 h after intravenous injection of HTiO_2_ NPs (5 mg/kg). NIR fluorescence images of tumour were obtained with the small-animal imaging system using the Cy5.5 channel (λ_exc_ = 620–650 nm and λ_em_ = 680–710 nm).

### *In vivo* SDT with HTiO_2_ NPs in the liver tumour model

To evaluate the antitumour efficacy of HTiO_2_ NPs in the SCC7 liver tumour model, tumour-bearing mice were prepared as described above. After 3 days, HTiO_2_ NPs were injected into the tail vein of the mice and the mice were divided into three groups: (a) non-treated (3 days), (b) non-treated (10 days), and (c) HTiO_2_ NPs + US (10 days). Tumour-bearing mice were treated with US 3 h after intravenous injection. US treatment for liver tumour tissue was performed over a total period of 330 seconds (power: 30 W, frequency: 1.5 MHz, duty cycle: 10%, pulse repetition frequency: 1 Hz, time: 30 seconds, interval: 2 mm). *In vivo* ultrasound 3D rendering images were obtained using a Vevo770^®^ High-Resolution Micro-Imaging System (Visualsonics, Toronto, Canada) equipped with a RMV 706 probe (frequency: 40 MHz, power: 100%, RF cycles: 1, sound speed: 1540 m/second, depth: 1 mm, FOV: 10 × 10 mm, frame rate: 11 Hz, 3D step size: 0.102 mm). Results for tumour volume inhibition were obtained using the OV-100 small-animal imaging system. Tumour volumes were calculated as **a** × **b**^2^ × 0.54, where **a** is the largest and **b** the smallest diameter. *Ex vivo* images of major organ images were also obtained using the OV-100.

## Additional Information

**How to cite this article**: You, D. G. *et al.* ROS-generating TiO_2_ nanoparticles for non-invasive sonodynamic therapy of cancer. *Sci. Rep.* **6**, 23200; doi: 10.1038/srep23200 (2016).

## Supplementary Material

Supplementary Information
