# Supplementary material for: ROS-generating TiO2 nanoparticles for non-invasive sonodynamic therapy of cancer
Source: Sci Rep. 2016 Mar 21;6:23200. doi: 10.1038/srep23200 (PMC4800401; doi:10.1038/srep23200)
Supplement: Supplementary Information [file srep23200-s1.pdf]

(Manuscript for *Scientific Reports*)

ROS-generating TiO_2 nanoparticles for non-invasive sonodynamic therapy of cancer

Dong Gil You[1,2,*], V.G. Deepagan[3,*], Wooram Um[2, 4], Sangmin Jeon[1,2], Sejin Son[2], Hyeyoun Chang[2,5], Hwa In Yoon[2,6], Yong Woo Cho[6], Maggie Swierczewska[7], Seulki Lee[7], Martin G. Pomper[7], Ick Chan Kwon[2], Kwangmeyung Kim[2,5] & Jae Hyung Park[1,3,4]

[1]School of Chemical Engineering, Sungkyunkwan University, Suwon 440-746, Republic of Korea.

[2]Center for Theragnosis, Korea Institute of Science and Technology, 39-1 Hawolgok-dong, Seongbuk-gu, Seoul 136-791, Republic of Korea.

[3]Department of Polymer Science and Engineering, Sungkyunkwan University, Suwon 440-746, Republic of Korea.

[4]Samsung Advance Institute for Health Sciences and Technology, Sungkyunkwan University, Suwon 440-746, Republic of Korea.

[5]Korea University of Science and Technology, 113 Gwahangno, Yuseong-gu, Daejeon 305-333, Republic of Korea.

[6]Department of Chemical Engineering, Hanyang University, Ansan 426-791, Republic of Korea.

[7]The Russell H. Morgan Department of Radiology and Radiological Science, Johns Hopkins School, Baltimore, Maryland 21287-0006, United States.

* These authors contributed equally to this paper.

Corresponding authors:

Jae Hyung Park, Ph.D.
Tel: +82-31-290-7288; fax: +82-31-299-6857; e-mail: jhpark1@skku.edu

Kwangmeyung Kim, Ph.D.
Tel: +82-2-958-5916; fax: +82-2-958-5909; e-mail: kim@kist.re.kr

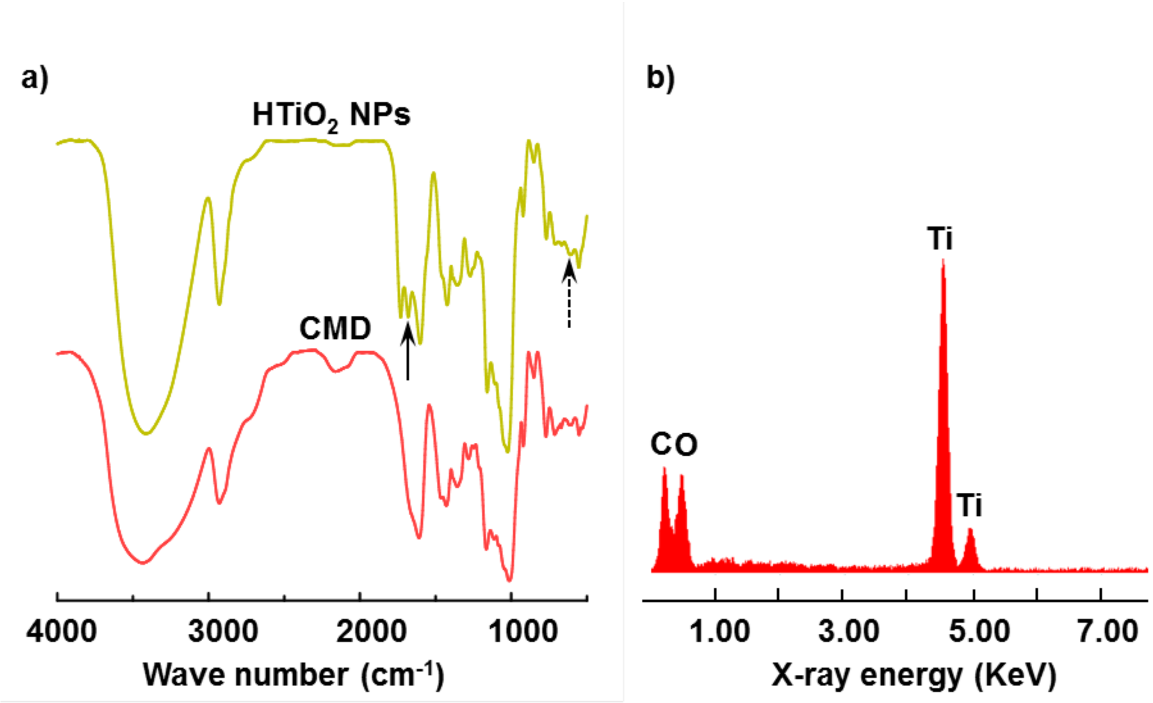

Figure S1. (a) FT-IR spectra of $HTiO_2$ NPs and CMD. The peak at 600 cm^{-1}, indicated by a dashed arrow, is attributed to C-C bending vibration of dopamine. The peak at 1686 cm^{-1}, indicated by a solid arrow, is assigned to the carbonyl stretching vibration of the amide bond between CMD and dopamine. (b) EDS spectrum of $HTiO_2$ NPs.

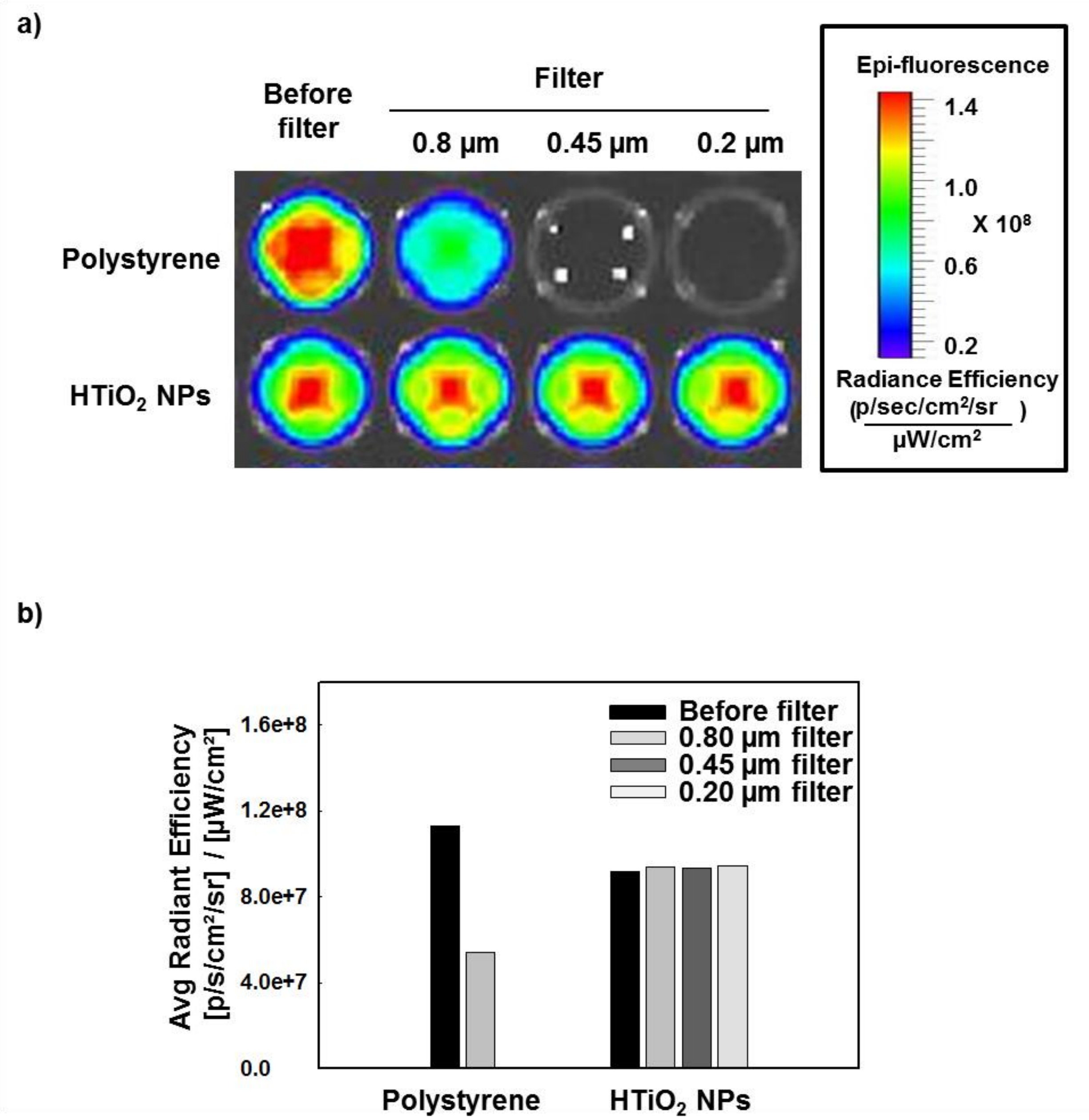

Figure S2. Flexibility of $HTiO_2$ NPs. (a) Fluorescence image of $HTiO_2$ NPs after passing through a series of syringe filters. (b) Quantification of fluorescence signal intensity.

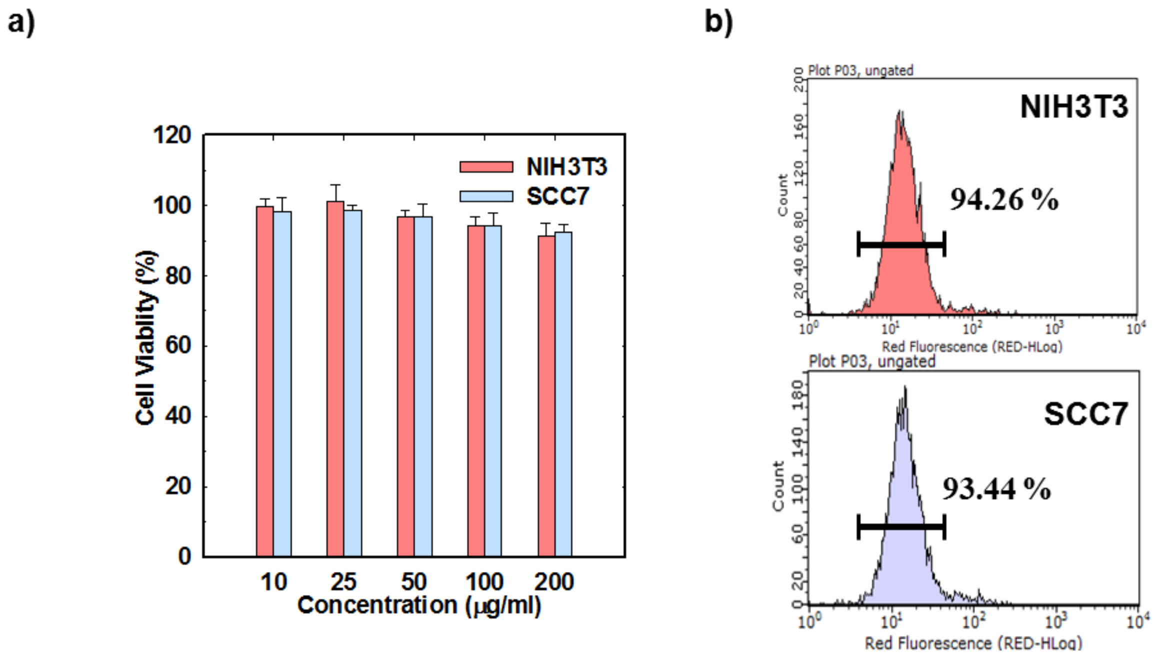

Figure S3. Toxicity of $HTiO_2$ NPs. (a) *In vitro* cytotoxicity of $HTiO_2$ NPs, measured by the MTT assay and (b) FACS analysis.

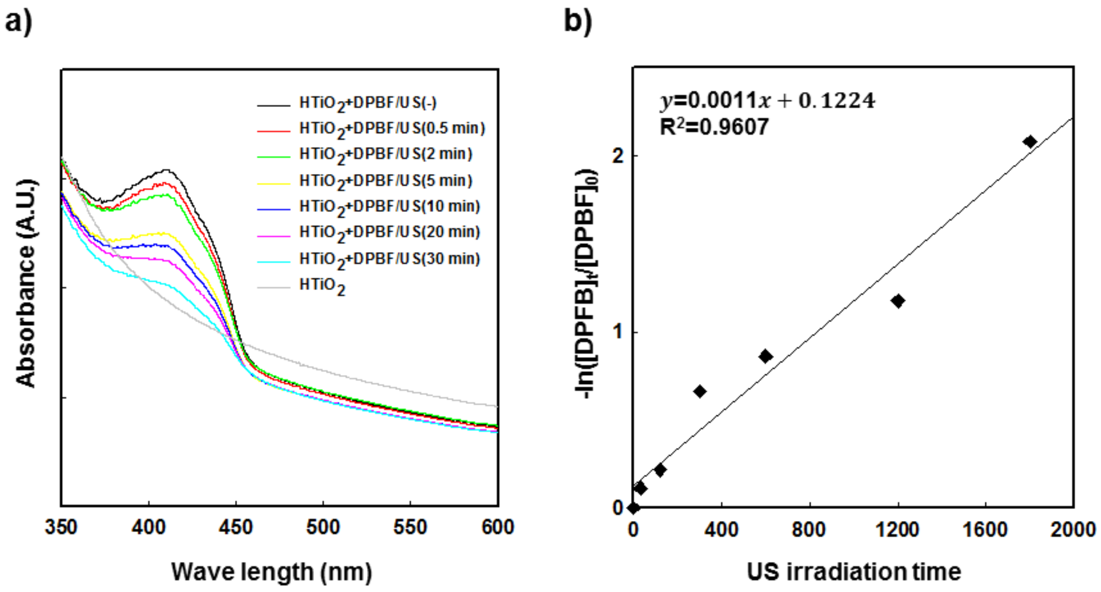

Figure S4. The singlet oxygen generation ability of $HTiO_2$ NPs with ultrasound. (a) UV-Vis spectra of DPBF with increasing exposure time. (b) First-order plot of DPBF absorbance versus time.

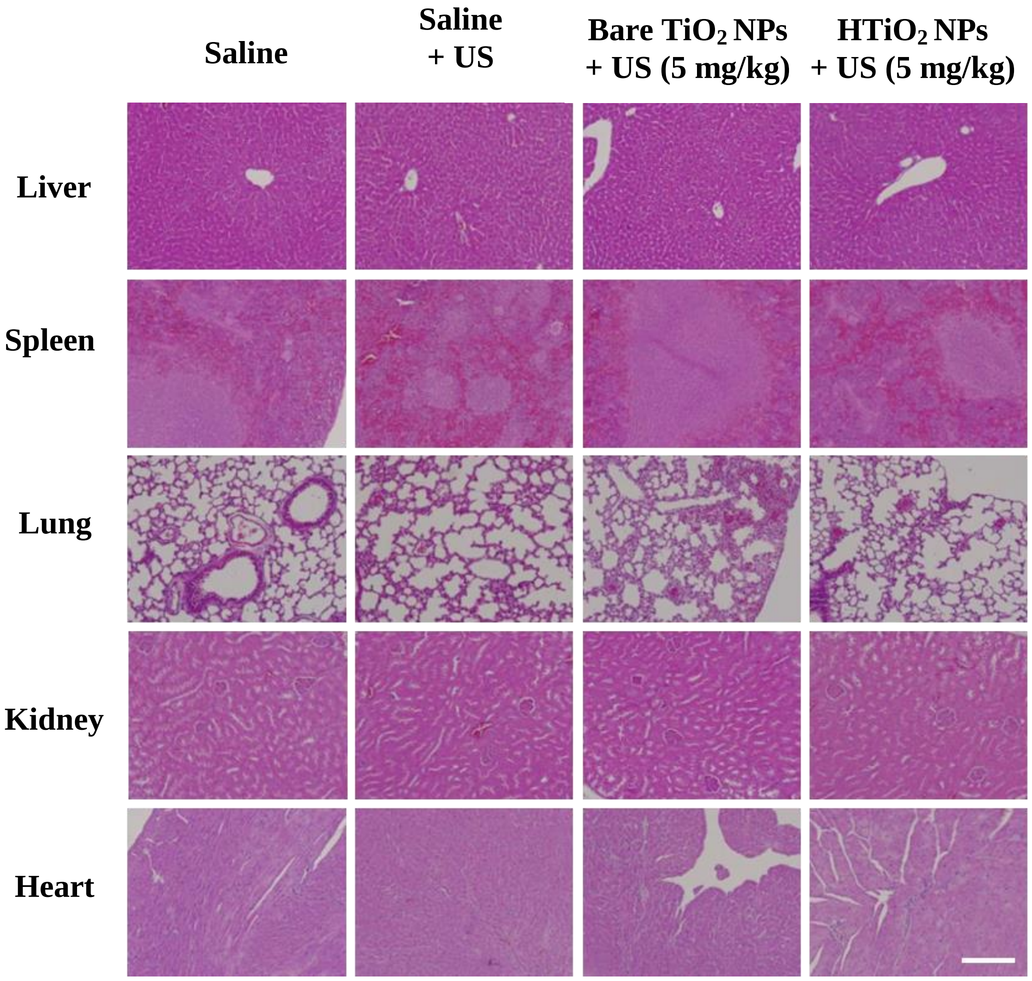

Figure S5. H&E staining of major organs after SDT. (Scale bar, 200 μm).

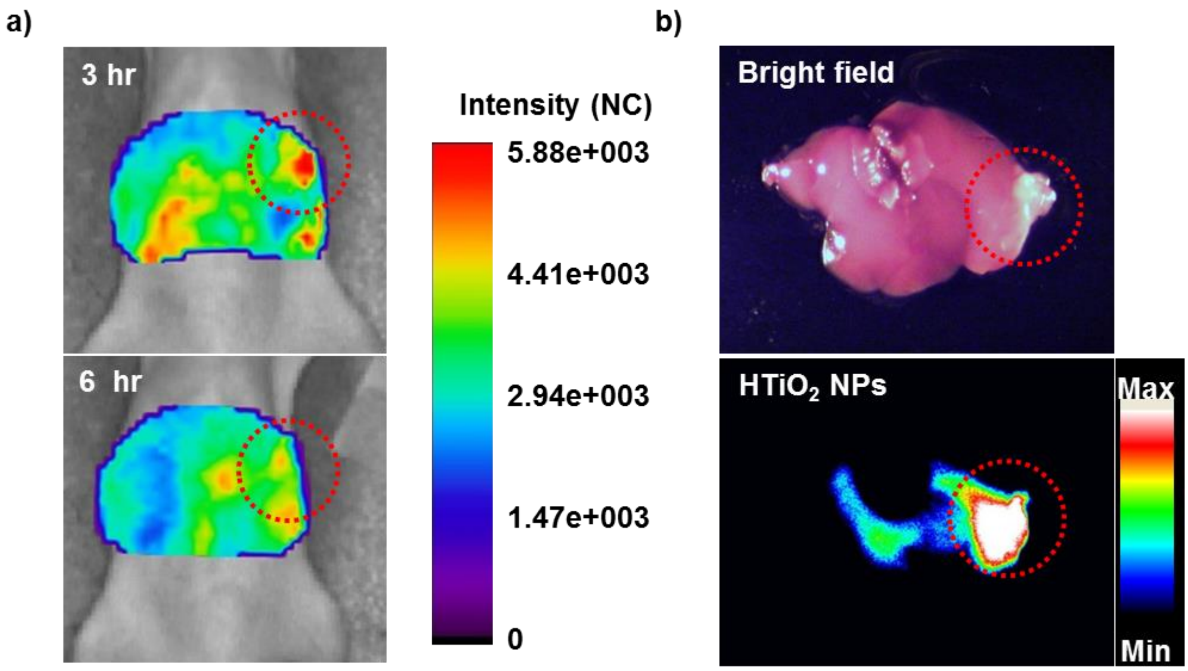

Figure S6. *In vivo* biodistribution of $HTiO_2$ NPs in the liver tumor model. (a) Time-dependent accumulation of nanoparticles in mice bearing liver tumor after intravenous injection of $HTiO_2$ NPs. (b) *Ex vivo* bright-field and fluorescence images of liver tumor 6 h post injection. The doted circle indicating the tumor region.
